# Pneumococcal carriage among children under five in Accra, Ghana, five years after the introduction of pneumococcal conjugate vaccine

**DOI:** 10.1186/s12887-019-1690-5

**Published:** 2019-09-05

**Authors:** Nicholas T. K. D. Dayie, Elizabeth Y. Tettey, Mercy J. Newman, Elizabeth Bannerman, Eric S. Donkor, Appiah-Korang Labi, Hans-Christian Slotved

**Affiliations:** 1 Dept. of Medical Microbiology, School of Biomedical and Allied Health Sciences University of Ghana, Accra, Ghana; 2 Department of Bacteria, Parasites and Fungi, Statens Serum Institut, Artillerivej 5, DK-2300 Copenhagen, Denmark

**Keywords:** *Streptococcus pneumoniae*, Ghana, Carriage, Serotype, PCV-13

## Abstract

**Background:**

The study objective was to determine the carriage and serotype distribution of *Streptococcus pneumoniae* among children in Accra, Ghana, five years after the introduction of the pneumococcal conjugate vaccine (PCV-13) in 2012.

**Methods:**

Nasopharyngeal swab samples were collected from 410 children below 5 years of age in Accra, Ghana, from September to December, 2016. Pneumococcal isolates were identified by optochin sensitivity and bile solubility. Serotyping was performed using the latex agglutination kit and Quellung reaction. The isolates were furthermore tested for antimicrobial susceptibility for different antimicrobials, including penicillin (PEN). Twelve isolates including seven non-typeable (NT) isolates were characterized using whole-genome sequencing analysis (WGS).

**Results:**

The overall carriage prevalence was found to be 54% (95% CI, 49–59%), and 20% (95% CI, 49–59%) of the children were carrying PCV-13 included serotypes, while 37% (95% CI, 33–42%) of the children were carrying non-PCV-13 serotypes. Based on the serotype distribution, 33% of all observed serotypes were included in PCV-13 while 66% were non-PCV-13 serotypes. The dominating non-PCV-13 serotypes were 23B, 16F, and 11A followed by PCV-13 serotypes 23F and 19F. The PCV-13 covers the majority of resistant isolates in Accra. A proportion of 22.3% of the isolates showed intermediate resistance to penicillin G, while only one isolate showed full resistance. Forty-five isolates (20.5%) were defined as multidrug-resistant (MDR) as they were intermediate/resistant to three or more classes of antimicrobials. Of the seven NT isolates characterized by WGS, four showed highest match to genotype 38, while the remaining three showed highest match to genotype 14. Four MDR serotype 19A isolates were found to be MLST 320.

**Conclusion:**

PCV-13 introduced in Ghana did not eliminate PCV-13 covered serotypes, and the carriage rate of 54% in this study is similar to carriage studies from pre PCV-13 period. However, the penicillin non-susceptible isolates have been reduced from 45% of carriage isolates before PCV-13 introduction to 22.3% of the isolates in this study. Continuous monitoring of serotype distribution is important, and in addition, an evaluation of an alternative vaccination schedule from 3 + 0 to 2 + 1 will be important to consider.

**Electronic supplementary material:**

The online version of this article (10.1186/s12887-019-1690-5) contains supplementary material, which is available to authorized users.

## Background

*Streptococcus pneumoniae* (pneumococcus) is considered the leading pathogen associated with community-acquired pneumonia, otitis media and meningitis [1]. Pneumococcal appearance in humans can be divided into two phases, carriage and the disease phase, where the carriage of *S. pneumoniae* is generally described as the prerequisite for developing pneumococcal infections, and often young children are considered to act as reservoirs [2, 3]. Pneumococcal infections have attracted global public health attention due to the high burden of disease and associated mortality, particularly among children under five and adults > 64 years in resource poor countries [1, 4, 5]. The high burden of morbidity and mortality associated with the pneumococcus can be reduced using appropriate vaccines. Hence, recently attention has been given to the introduction of pneumococcal conjugate vaccines into the children vaccination programmes in the developed and developing countries [1, 5, 6].

Since 2000, studies have shown that the introduction of the pneumococcal conjugate vaccines (PCV7, PCV-10 and PCV-13 in selected countries) has been effective particularly among children under 5 years [1, 7–10]. Based on these results, the vaccines have been introduced in other parts of the world including Africa [5, 6, 11].

However, to be able to measure the impact of PCVs, it is important to have pre-vaccination data on the serotype distribution [10–12]. Several studies from Africa prior to the introduction of PCVs have been performed and showed that the major serotypes were 1, 5, 6A, 6B, 14, 19A, 19F and 23F [4, 10, 11, 13]. In Ghana, the PCV-13 was introduced as part of the routine childhood immunization programme in May 2012, using the 3 + 0 vaccination schedule [6, 13]. The official country report on PCV13 coverage was estimated to be 99% in 2017 (http://www.view-hub.org, accessed 02-09-2019). Prior to the PCV-13 introduction in Ghana, several studies showed the nature and distribution of pneumococcal serotypes circulating in Ghana [13–16]. The carriage study by Dayie et al. [13] showed that the predominant serotypes were 19F, 6B, 23F and 6A, and a PCV-13 vaccine coverage was estimated to be approximately 50%. Other studies have shown that the introduction of the PCV in the routine childhood immunization programme has reduced the carriage of vaccine serotypes but there has been an increase in non-PCV serotypes [2, 10]. Studies from the Gambia showed that PCV-7 and PCV-13 had a positive effect on the vaccine-type carriage (VT-carriage), while an increase in the carriage prevalence of non-PCV serotypes was observed [5, 10]. Five years after the introduction of the pneumococcal conjugate vaccine in Ghana, there is no post PCV-13 data on prevailing circulating serotypes to measure the impact of PCV-13 among the healthy Ghanaian population. In addition, the previous study by Dayie et al. [17] showed an increasing incidence of multidrug resistant pneumococci among carriage isolates; hence, data to determine the impact of PCV-13 vaccinations and the trend of antibiotic resistance in pneumococci among children under five is needed.

The aim of this study was to determine the pneumococcal serotype distribution and antimicrobial susceptibility patterns of carriage isolates among healthy children (≤ 5 years), five years post PCV-13 vaccination in Accra, Ghana,.

## Methods

### Study sites

The study was carried out in the Accra metropolis, which is the capital city of Ghana and falls within the coastal belt with humid and warm climatic conditions. Accra has the second highest population density compared to other districts in Ghana (https://www.indexmundi.com/ghana/demographics_profile.html; accessed 02-09-2019). The PCV-13 is part of the routine childhood immunization programme in Ghana, and the vaccination schedule is 6, 10 and 14 weeks [13].

### Sampling and study design

The study was carried out in nurseries and kindergartens within the Accra metropolis of the Greater Accra region of Ghana from September to December 2016.

A list of nurseries and kindergartens in the Accra metropolis was obtained from the Ghana Education service. Seven schools were randomly selected and written consent was obtained from the parents of the children. Children whose parents declined to give their consents were excluded from the study; children who declined assent after parental consent were also excluded. Children with active upper respiratory tract infections or who had been given antibiotics within the last two weeks prior to sampling were excluded. Postnatal cards were obtained from the parents in order to ascertain the vaccination status of the children.

### Specimen collection

Nasopharyngeal specimens were collected using a WHO recommended methodology [16]. From September to December 2016, nylon-tipped paediatric sized FlOQSwabs (Copan Flock Technologies, Italy) were used to collect nasopharyngeal specimens. Four hundred and ten swab samples were obtained. Immediately after collection the swab specimens were placed in premade vials containing 1 ml of skim milk-tryptone-glucose-glycerin (STGG) medium and transported on ice to the laboratory within 3 h of collection. Upon arrival at the laboratory the swab samples were immediately stored at -80 °C pending further processing [18].

### Characterization of *S. pneumoniae*

The specimens were processed based on the WHO recommendation for characterizing *S. pneumoniae* [18]. The samples were inoculated onto a 5% sheep blood agar containing 5 μg/ml of Gentamicin and then incubated at 37 °C in 5% CO_2_ for 18-24 h. A representative number of alpha-haemolytic colonies were subjected to optochin susceptibility testing, and based on the visual evaluation and the isolates’ susceptibility to optochin (inhibition zone ≥14 mm), swab samples were identified as containing possible *S. pneumoniae*. All swab samples suspected to contain *S. pneumoniae* isolates were transported on dry ice to Statens Serum Institut (SSI), Copenhagen, Denmark for further characterization. At SSI, the organisms were isolated from the swab samples and verified as pneumococcal isolates using phenotypic methods as described in previous studies [2, 19]. Briefly, 10 μl of the swab samples were cultured in serum broth overnight, the following day 1 μl of each serum broth was cultured on 10% horse blood agar plates and incubated overnight at 37 °C, 5% CO2. All serum broths were screened for multiple serotypes by using the Pneumotest-latex agglutination kit (SSIDiagnostica, Denmark) [2]. Serotyping/grouping of the isolates was performed using the Pneumotest-latex agglutination kit (SSIDiagnostica, Denmark) and the results were confirmed by the Quellung reaction test using the serotype specific antisera (SSIDiagnostica, Denmark) [19]. Non-typeable strains were defined as isolates presenting no phenotypic detectable capsule.

### Characterization of selected isolates

Due to financial constraints, we were only able to perform whole genome sequencing (WGS) on seven of the ten NT isolates and five of the multidrug-resistant (MDR) isolates. WGS was performed on 12 isolates. The isolates were sequenced by paired-end Illumina sequencing. Genomic DNA was extracted using a DNeasy Blood & Tissue Kit (QIAGEN, Hilden, Germany) and fragment libraries were constructed using a Nextera XT Kit (Illumina, Little Chesterford, UK) followed by 250-bp paired-end sequencing (MiSeqTM; Illumina) according to the manufacturer’s instructions. The paired-end Illumina data were de novo assembled using CLCbio’s Genomics Workbench v.7.5 QIAGEN) reporting only contigs > 500 bp using standard settings.

Bioinformatics, including blast, was done using the software CLC Main Workbench (Version 7.9.1, www.qiagenbioinformatics.com).

Multilocus Sequence Analysis (MLSA) as described by Bishop et al. [20], and the presence of cytosine at the 203 position using the 16S rRNA sequence [21] confirmed the pneumococcal species identification for all 12 isolates. The presence/absence of a gene was based on a cut-off of 80% coverage and a 95% identity for positive gene detection in this study [22].

The presence of capsular genes for all 12 isolates were blasted for 92 capsular polysaccharide genes (CPS genes) as described by Kapatai et al [22].

Multilocus sequence typing (MLST) was performed using the PubMLST DataBase (https://pubmlst.org/spneumoniae/) to identify the sequence type (ST) for each of the isolates.

The isolates were also analyzed for their Penicillin-Binding Protein (PBP) signature, based on a genotyping proposal and algorithm described for PBP1A, PBP2B and PBP2X [23], where the combination of the three PBP signatures determines the level of beta-lactam resistance. The isolates were tested by blast with the published types of predictive mutations vs. resistance levels of PBP1A, PBP2B and PBP2X proteins as described in Li et al. [23] and CDC (https://www.cdc.gov/streplab/pneumococcus/mic.html, accessed 18-06-2019).

Also, the presence of the genes *ermB and tet* were tested, and ResFinder 3.0 (https://cge.cbs.dtu.dk/services/ResFinder/) (80% ID threshold and 60% minimum length settings) was used to confirm the presence of the three genes [24].

### Antimicrobial susceptibility testing

Penicillin susceptibility testing was initially determined by agar-disc diffusion using 1 μg oxacillin disc (Oxoid Company, UK). Minimum inhibitory concentrations (MICs) for all oxacillin resistant isolates (R < 20) were determined using penicillin G MIC strips (Oxoid Company, UK).

Penicillin (PEN) susceptibility was defined as susceptible (MIC ≤0.06 μg/ml), intermediate (> 0.06–2 μg/ml) and resistant (> 2 μg/ml) according to the European Committee on Antimicrobial Susceptibility Testing (EUCAST) guidelines with *S. pneumoniae* ATCC 49619 used as a control (EUCAST Clinical Breakpoint Tables v. 6.0, valid from 2016 to 01-01).

All isolates were further tested using the disc diffusion method against erythromycin (ERY) (15 μg disk), tetracycline (TET) (30 μg disk), trimethoprim-sulphamethoxazole (SXT) (1.25/23.75 μg disk) and levofloxacin (LEV) (5 μg disk). The susceptibility test using Oxoid disks (Oxoid Company, UK) was performed by spreading an inoculum of 0.5 McFarland standard onto Müller-Hinton (Oxoid, UK) agar plates containing 5% sheep blood. The plates were incubated between 18 and 24 h at 37 °C in a 5% CO_2_ incubator, after which the zones of inhibition were measured with a calliper.

Multidrug-resistant (MDR) isolates are defined as isolates showing resistance (intermediate or resistant) to at least three classes of antimicrobials [9].

### Data analysis

Data were analyzed using Graph Pad Prism version 7 (GraphPad Software) for descriptive statistical analysis. R version 3.5.0 (2018-04-23) was used for calculation of confidence intervals (95% CI) and for the logistic regression model using the glm function in R (R version 3.5.0 (2018-04-23) for calculations in the univariable and multivariable model. *P*-value < 0.05 was considered significant.

## Results

### Characteristics of the study group

Four hundred and ten children participated in the study with almost an equal distribution of gender (52.5% were male). The mean age of the group was 39 months with a range of 6 months to 60 months of age. Four hundred and seven (407) of the 410 children were fully vaccinated with three doses of PCV-13 vaccines, two were of unknown vaccination status; one child was unvaccinated. All children with detected carriage were vaccinated with three doses of PCV-13 (Table 1).

**Table 1:** Characteristics of participating children

	Total number of children	Number of children with carriage of *S. pneumoniae* (%, 95 CI)	OR (95% CI)* (*p*-value)*	
Overall carriage rate	410	220 (54, 49–59)		
Females carriage rate	200	108 (54, 47–61)	1	
Males carriage rate	210	112 (53, 47–60)	0.97 (0.66–1.44) (*P* = 0.892)	
Number of Children carrying PCV-13 serotypes^a^	410	81 (20, 16–24)		
Number of Children carrying non-PCV-13 serotypes^a^	410	153 (37, 33–42)		
	Median age (Month)	Range (Month)	Interquartile range (month)	
Children	36	(6–60)	36–48	
Males	36	(6–60)	36–48	
Females	36	(12–60)	36–48	
	Vaccination status of all participants	Vaccination status of carriers		
PCV13 vaccinated	407 (99.3%)	220 (100%)		
Unknown	2	0		
Not vaccinated	1	0		
Age group (months)	Total number of children	Number of children with carriage of *S. pneumoniae* (%) (95% CI)	OR (95% CI)* (*p*-value)*	Number of children with multiple serotypes
0–11	2	1 (50, 13–200)	1	0
12–23	22	9 (40, 25–68)	0.69 (0.03–19.06) (*P* = 0.804)	0
24–35	73	41 (56, 46–69%	1.28 (0.05–33.24) (*P* = 0.863)	3
36–47	178	105 (59, 52–67%)	1.44 (0.05–36.76) (*P* = 0.798)	10
48–60	135	64 (47) (40–57%)	0.90 (0.03–23.11) (*P* = 0.942)	1
Total	410	220 (54) (49–59%)		14

*Odds ratios, confidence intervals and *p*-values were calculated using a generalized linear model (glm function in R)

a. Because 14 children were carrying two different serotypes, the number of children carrying the multiple serotupes in these two groups exceed 220 children and the figure is currently 234 cases

### Carriage rate and serotype distribution

The observed overall carriage rate was 54% (95% CI, 49–59%) with nearly identical carriage rate between male and female children (Table 1). Because there was no difference in carriage by sex, only the OR and not adjusted OR was calculated for each age group (Table 1). The age group 36–47 month showed the highest carriage rate of 59% (95% CI, 52–67%) (Table 1). Two hundred and thirty four pneumococcal isolates were isolated from 220 children of which 14 children harbored two different pneumococcal serotypes (Table 1).

**Fig. 1:**
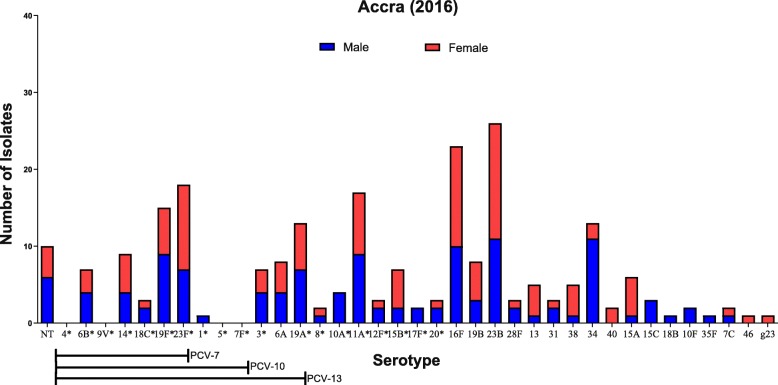
Serotype distribution of *S. pneumoniae*, by gender, in children ≤60 month of age, in Accra, Ghana. The serotypes are listed on the X-axis, starting with the NT, and followed by the serotypes covered by PCV-7, PCV-10 and PCV-13 vaccines. g23: one isolate was only determined to belong to group 23. * Serotypes covered by PPV-23

The predominant serotypes observed were the non-vaccine serotypes 23B (11%) and 16F (10%) followed by the vaccine serotypes 23F (8%) and 19F (6%). The dominating PCV-13 serotypes were 23F, 19F, 19A (6%) while non-PCV-13 serotypes were 23B, 16F, 11A (7%), and 34 (6%) (Fig. 1). Ten isolates were found to be non-typeable isolates (Table 1).

34.6% of the detected serotypes were covered by the PCV-13, and 65.4% of the isolates (including NT isolates) were found to be non-PCV-13 serotypes (Table 2).

**Table 2:** Distribution of *S. pneumoniae* isolates with intermediate/full resistance towards four antimicrobials by vaccine coverage. All isolates were sensitive to Levofloxacin

	Number of isolates tested for susceptibility	Number of non-susceptible isolates (% of all isolates)	Number of non-typable tested for susceptibility	Number of non-susceptible PCV-13 serotypes	Number of non-susceptible non-PCV13 serotypes
All isolates	220	191 (86.8%)	10 (4.5%)	76 (34.5%)	144 (65.5%)
PEN (MIC 0.06–2)	220^a^	49 (22.3%)	7 (3.2%)	27 (12.3)	13 (6.0%)
PEN (MIC > 2)		1 (0.5%)	0	0	1 (0.5%)
TET (25 > I > =22)	219^b^	2 (0.9%)	0	1 (0.5%)	1 (0.5%)
TET (R < 22)		138 (63.0%)	7 (3.2%)	64 (29.2%)	51 (2.3%)
SXT (18 ≥ I ≥ 15)	220^c^	32 (14.5%)	1 (0.5%)	10 (4.5%)	18 (8.2%)
SXT (R < 15)		135 (61.4%)	8 (3.6%)	58 (26.4%)	43 (19.5%)
ERY (S ≥ 22 > I ≥ 19)	220^d^	11 (5.0%)	1 (0.5%	4 (1.8%)	5 ((2.3%)
ERY (R < 19)		24 (10.9%)	0	15 (6.8%)	8 (3.6%)
MDR ≥ 3	220	45 (20.5%)	4 (1.8%)	27 (12.3)	18 (8.2%)

^a^Information on Penicillin susceptibility for 14 isolates is not available

^b^Information on Tetracycline susceptibility for 15 isolates is not available

^c^Information on SXT susceptibility for 14 isolates is not available

^d^Information on Erythromycin susceptibility for 14 isolates is not available

In Additional file 1: Table S1, the serotype distribution data from Dayie et al. [2013] and the data from the present study has been presented, thereby making it possible to compare the data set from the two carriage studies.

### Antimicrobial resistance

22.3% of the isolates showed intermediate resistance to penicillin G, while one isolate showed penicillin resistance (Table 2).

**Fig. 2:**
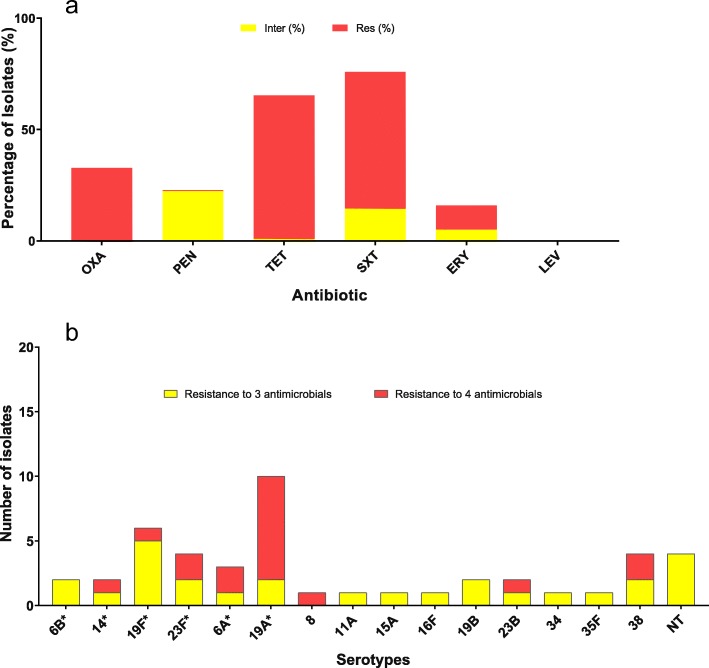
The antimicrobial susceptibility and serotype distribution of Multidrug-resistant (MDR) defined isolates. MDR isolates are defined as isolates showing resistance (intermediate or resistant) to at least three classes of antimicrobials. Figure 2a presents the MDR ≥ 3, and Fig. 2b presents the total resistant isolates. ^a^Information on Penicillin susceptibility for 14 isolates is not available. ^b^Information on Tetracycline susceptibility for 15 isolates is not available. ^c^Information on Trimethoprim-sulphamethoxazole susceptibility for 14 isolates is not available. ^d^Information on Erythromycin susceptibility for 14 isolates is not available

The highest number of resistant isolates was observed for tetracycline (63%) and trimethoprim-sulphamethoxazole (61.4%) of which more than half of the strains showed resistance. In addition, two isolates were tetracycline intermediate resistant while 14.5% were trimethoprim-sulphamethoxazole intermediate resistant. It was observed that 11% of the isolates were resistant to erythromycin while 5% of the isolates were intermediate resistant (Table 2, Fig. 2). All isolates were sensitive to levofloxacin. 65.5% (144 isolates) of intermediate/resistant isolates were serotypes not included in the PCV-13 (Table 2).

Twenty-seven isolates (12.3%) were found to be intermediate/resistant to three classes of antimicrobials, while 18 isolates (8.2%) were intermediate/resistant to four classes of antimicrobials (Fig. 2). In total 45 isolates (20.5%) were defined as MDR isolates as they were intermediate/resistant to three or more classes of antimicrobials. Twenty-eight isolates of the 45 MDR isolates were covered by the PCV-13 vaccine (Fig. 2).

### Molecular characteristics of 12 isolates

Four of the seven analyzed NT isolates showed a preference for genotype 38 showing the highest hit score (Table 3). Three of these isolates (G10, C131 and C28) showed identical MLST presenting a novel ST within the Clonal Complex ST908 (the seven loci *ddl* was unidentified) (Table 3). The fourth isolate (G11) was ST344, and was completely different from the other three isolates. The remaining three NT isolates showed the highest hit score for genotype 14 (Table 3). Two of the isolates G7 with ST (2–14–37-36-29-17-21) and G140 with ST9735 were relatively closely related with only one locus difference (the *recP* locus). The third isolate (C139) was not related to the two other isolates (G7 and G140).

**Table 3:** Twelve isolates were analyzed by WGS. The 12 isolates consisted of seven non-typeable isolates and five MDR defined isolates

Isolate number	MLST type (ST)^a^	Clonal complex (CC)/ nearest ST	Genotype/Capsular locus top hit (Nearest match)	Serotype	Pen/Tetra/SXT/Ery (Phenotypical)	*tet*(M)	*ermB*	PBP 1a	PBP 2b	PBP 2x	Predicted value*/Phenotype value
G10	(2–5–36-12-2-21-?)	ST908/ST11041	Top hit: Genotype 38	NT	I/S/R/S	–	–	38	25	43	MIC =? / MIC = 1
C131	(2–5–36-12-2-21-?)	ST908/ST11041	Top hit: Genotype 38	NT	I/S/R/S	+ (Tn*916*)	–	38	25	43	MIC =? / MIC = 2
C28	(2–5–36-12-2-21-?)	ST908/ST11041	Top hit: Genotype 38	NT	I/S/R/S	+ (Tn*916*)	–	38	25	43	MIC =? / MIC = 2
G11	344	Singleton	Top hit: Genotype 38	NT	I/R/R/S	+ (0)^c^	–	55	16	150	MIC =? / MIC = 0.12
C139	(2–149–1-12-6-494-14)	ST11264	Top hit: Genotype 14	NT	S/R/I/S	+ (Tn*916*)	–	24	73	192	MIC =? / Oxa sentitive
C140	9735	CC63	Top hit: Genotype 14	NT	S/R/R/S	+ (Tn*916*)	–	24	73	192	MIC =?/Oxa sentitive
G7	(2–37–36-29-17-21-14)	ST9735	Top hit: Genotype 14	NT	I/R/R/S	+ (Tn*916*)	–	24	73	192	MIC =? / MIC = 0.12
D012	2613	Singleton	Genotype 15A	15A	I/R/S/R	+ (Tn*916*)	+ (0)^c^	34	89	147	MIC = 2/ MIC = 2
G14	320	CC320	Genotype 19A	19A	I/R/R/R	+ (0)^c^	+ (0)^c^	13	11	16	MIC = 4/ MIC = 1
G27	320	CC320	Genotype 19A	19A	I/R/R/R	+ (0)^c^	–	13	11	16	MIC = 4/ MIC = 1
G28	320	CC320	Genotype 19A	19A	I/I/I/R	+ (0)^c^	+ (0)^c^	13	11	16	MIC = 4/ MIC = 1
C88	320	CC320	Genotype 19A	19A	I/R/R/R	+ (0)^c^	+ (0)^c^	13	11	16	MIC = 4/ MIC = 1

^a^The MLST locus allele is presented as (*aroE*-*gdh*-*gki*-*recP*-*spi*-*xpt*-*ddl*)

* Predicted level of resistance to penicillin according to CDC (https://www.cdc.gov/streplab/pneumococcus/mic.html)

? Predicted value is not presented (https://www.cdc.gov/streplab/pneumococcus/mic.html)

With the five tested MDR isolates, the serotypes were confirmed by the genotypes and STs (Table 3).

Four serotype 19A isolates (G14, G27, G28, C88) were found to be MDR isolates. All four isolates were ST 320 and showed the same PBP profile (13, 11, 16). The PBP profile corresponded to the phenotypic susceptibility profile as penicillin intermediate (Table 2). A fifth serotype 15 MDR isolate (D012) also showed a PBP profile corresponding to the phenotypic susceptibility profile.

Eleven isolates harbored the *tet*(M) gene and four isolates harbored the *ermB* gene according to ResFinder 3.0 (Table 3). The presence of *ermB* gene was generally in agreement with the phenotypic antibiotic susceptibility result, while two of the isolates (C131, C28) harboring the *tet*(M) gene, were still found to be phenotypically sensitive (Table 3). Based on the information from the resistance gene, none of the isolates harboring the *ermB* gene were found positive for the presence of mobile genetic elements of Tn-family, while Tn917 was found in six isolates harboring the *tet*(M) gene. The two isolates harboring the *tet*(M) gene, but still found phenotypical sensitive also showed presence of the transposon Tn916 (Table 3).

## Discussion

Worldwide pneumococcal carriage studies have been performed to measure the impact of the PCV vaccination among children [5, 10, 25, 26]. The majority of carriage studies performed in Africa were baseline studies with the purpose of evaluating the effectiveness of the PCVs [5, 12, 25–27]. This study is to our knowledge the first carriage study performed in Accra, Ghana, among healthy children to evaluate the effect of PCV-13 on pneumococcal carriage five years after the PCV-13 introduction [13]. Only few other studies in the region have performed post PCV introduction carriage studies [5, 10].

The overall carriage rate observed in this study after five years of PCV-13 vaccination was 54% (95% CI, 49–59) (Table 1). Two pre-PCV-13 carriage studies from Accra performed in 2011, both on healthy children below 5 years of age, showed a carriage rate of 34% in nursery/kindergarten children [13] and a 49% carriage rate in children from a pediatric hospital in Accra [16]. Comparing the carriage rate for PCV-13 serotypes (18%) and the non-PCV-13 serotypes (19%) from the pre-PCV vaccination period from Accra [13] with the carriage rate observed in this study for PCV13 serotypes (20, 95% CI, 16–24) and the carriage rate for non-PCV serotypes (37, 95% CI 33–42) in Accra, show that the PCV13 carriage rate have not changed or increased, while an increase in the non-PCV serotypes carriage rate was observed. The PCV-13 introduction does therefore not seem to have had a reducing effect on the overall carriage rate in children in Accra, Ghana. Other studies have also observed no net effect of the carriage rate after PCV introduction [27]. In the Gambia, they observed no net effect on the carriage rate after 2 years of PCV-7 vaccination [5] and after five years of PCV-13 vaccination [10]. A Danish study also observed that the overall carriage rate in children was not reduced after more than 10 years of PCV vaccination [2].

Before the introduction of the pneumococcal vaccination in Ghana, the PCV-13 showed a coverage of 48% of the detected carried serotypes [13], while in this study the coverage rate of the PCV-13 was 35%. The introduction of the PCV-13 in Ghana has had an effect on the serotype distribution although both the overall carriage rate has not been reduced and PCV-13 serotype carriage rate are still the same (between 18 and 20%) [13]. VT-serotypes are, however, still found as carriage serotypes in the vaccinated children in this study. This has also been observed in the study from the Gambia [10] while it was not the case in a Danish carriage study, where VT serotypes rarely were detected [2]. A possible explanation of this difference might be the vaccination schedule [10], which in the Gambia and Ghana is 3 + 0, while it is 2 + 1 in Denmark [2, 10].

Comparison of the serotype distribution observed in this post PCV-13 study in Ghana with the pre PCV-13 study in 2011 [13] showed that some of the VT-serotypes are still dominating, such as serotypes (6B, 14, 19F and 23F). However, changes in the serotype distribution have been observed, as the carriage prevalence of serotypes 6B and 19F in 2011 were 10 and 15%, respectively, of the detected isolates in Accra, while in 2017, the carriage prevalence of serotypes 6B and 19F were 3 and 6%, respectively. An increase in carriage prevalence was observed for serotype 19A from about 1.3% in 2011 to 5.6% in 2017. Generally, none of the PCV-13 serotypes in 2017 was found to be more than 8% of the total serotypes observed.

The overall predominant serotypes in 2017 were serotype 23B and serotype 16F, which are not included in either PCV-13 or PPV-23. In addition, serotype 11A (not included in PCV-13) and serotype 34 (not included in PCV-13 and PPV-23) were common serotypes. Of the four non-PCV serotypes, only 11A is included in the PPV-23, while the three other serotypes are not part of PPV-23 [13]. Although carriage studies cannot provide information on which new replacement serotypes might be the future dominant cause of pneumococcal disease, it can indicate whether the vaccine coverage might continue to be low with regard to detected serotypes [28], and a new PCV vaccine may have limited impact on the pneumococcal epidemiology in Ghana.

The carriage prevalence that was observed between age groups in this study (Table 1) was very similar to both the pre-PCV-studies, where the carriage rates peaked around age groups 24–35 months and 36–47 months [13] and around 43–48 months [16]. Because this study included only a limited number of children younger than 1 year, we cannot describe the effect of the PCV-13 vaccination carriage in this age group. However, other studies in Africa have shown that there is a high carriage also within this age group [5, 10, 29].

In the pre-PCV-carriage study from Ghana by Dayie et al. [13], 45% of the isolates from Accra showed penicillin intermediate resistance. Another carriage study from Ghana performed in 2011 also showed a high percentage of penicillin resistance of 63% [16]. In this study, we observed a decline in the prevalence of penicillin intermediate resistant isolates to 22% (Table 2).

In Ghana, the Standard Treatment Guidelines (Sixth Edition, 2010 – Ghana) recommend clinicians generally to use amoxiclav or the penicillin for pediatric infections (http://apps.who.int/medicinedocs/en/m/abstract/Js18015en/, accessed 02-09-2019). The decline in prevalence of penicillin intermediate resistance in this study may be attributed to the effect of PCV-13 vaccination, which was shown to cover more than half of the intermediate penicillin resistant isolates observed in the study by Dayie et al. [13].

While the PCV-13 vaccination seems to have reduced penicillin resistance in Ghana, this does not appear to be the case with tetracycline, which this study found to be about 63%, while previous pre-PCV studies have shown similar or higher tetracycline resistance of about 60–85% [16, 30]. In a carriage study in 2007, erythromycin resistance was not detected [30]; since then, several pre-PCV studies have, however, shown the presence of erythromycin resistance of up to 28% in Ghana [15–17]. In this study (Table 3, Fig. 2), 16% of erythromycin non-susceptible isolates were observed. Hence, it seems that erythromycin resistance has not changed greatly since the PCV-13 introduction.

Overall, we found that 20% of all the carriage isolates could be defined as MDR isolates, of which more than 60% of the serotypes were covered by the PCV-13 (Fig. 2). We furthermore observed a reduction of penicillin non-susceptible isolates covered by the PCV-13 compared to the study by Dayie et al. [13], and found that PCV-13 still covers most of the MDR isolates (Table 2). There is, therefore, still a possibility for great effect on reducing non-susceptible isolates with continuous PCV-13 vaccination.

Ten isolates were found to be non-typeable serotypes, of which we were able to perform WGS on seven of the non-typeable isolates (Table 3). The seven NT isolates were differentiated into only two possible genotypes, genotypes 38 and 14. One of the isolates, C140 with the ST 9735 showing a preferred genotype 14, also showed the same MLST type as ID 23690 (MLST database, https://pubmlst.org/). Isolate ID 23690 was a serotype 14 isolate submitted from the 2011 Ghana project [13]. This could support the relatedness of isolate C140 and isolate G7 to serotype 14 isolates, which have lost the ability to present the capsular gene for serotype 14.

Five of the MDR isolates were analyzed by WGS (Table 3). Four of the isolates of serotype 19A were found to belong to ST320 (CC 320), which is a well-known penicillin resistant serotype 19A clone that has been observed all around the world and in particular after the PCV-7 introduction in USA [31, 32]. All four 19A isolates also showed an identical PBP profile of 13–11-16, which is related to a penicillin MIC of 4 according to Li et al. [23].

The limitation of this study is that we did not include children that were < 11 month of age, who in other studies from Africa have been shown to have a high carriage rate [10, 29]. The study focused on children from nurseries and kindergarten, which do not include children below one year of age. However, by choosing this group of children we were able to compare to some extent the carriage prevalence found in the pre-PCV-13 study by Dayie et al. [13], in which the study subjects were also children from nurseries and kindergarten in Accra. Although, it also has to be mentioned that the pre-PCV-13 study by Dayie et al. [13] was conducted from March – July 2011 and this study was conducted from September to December 2016, which means that seasonal variation could be a possible factor that might have influenced carriage prevalence between the two studies. Nonetheless, regardless of the possible seasonal variability, it is our assertion that comparing the children in this study with the pre-PCV-13 study [13] have made it possible to see whether there had been any changes in the pneumococcal serotype distribution five years post-PCV-13 vaccination in Accra, Ghana.

## Conclusions

The introduction of PCV-13 in Ghana has reduced the carriage prevalence of serotypes covered by the PCV-13 although it has not removed them from the nasopharynx five years after the introduction of the vaccine. However, the PCV-13 vaccination covers majority of the non-antibiotic susceptible isolates. A further reduction of non-susceptible pneumococcal isolates is therefore within likelihood. Measuring the effect of PCV-13 vaccination by continuous monitoring of the serotype distribution is important to evaluate the effectiveness of PCV-13. In addition, an evaluation of an alternative vaccination schedule from 3 + 0 to 2 + 1 needs to be considered to obtain the full effect of PCV-13 vaccination.

## Additional file

Additional file 1: **Table S1.** The table presents the serotype distribution data from Dayie et al. [13] and the data from the present study, thereby making it possible to compare the data from the two carriage studies. (DOC 118 kb)

## Data Availability

The data and materials are available on request from the corresponding author (Hans-Christian Slotved, Ph.D., Senior Scientist, Department of Bacteria, Parasites and Fungi, Statens Serum Institut, Artillerivej 5, DK-2300 Copenhagen, Denmark, Tel: + 45 3268 8422, E-mail: hcs@ssi.dk.).

## Abbreviations

CPS genes: Polysaccharide genes
ERY: Erythromycin
EUCAST: European Committee on Antimicrobial Susceptibility Testing
LEV: Levofloxacin
MDR: Multidrug-resistant
MIC: Minimum inhibitory concentrations
MLSA: Multilocus Sequence Analysis
MLST: Multilocus sequence typing
NT isolates: Non-typeable isolates
PBP: Penicillin-Binding Protein
PCV-10: 10-valent pneumococcal conjugate vaccine
PCV-13: 13-valent pneumococcal conjugate vaccine
PCV-7: 7-valent pneumococcal conjugate vaccine
PEN: Penicillin
ST: Sequence type
STGG: skim milk-tryptone-glucose-glycerin medium
SXT: Trimethoprim-sulphamethoxazole
TET: Tetracycline
WGS: Whole genome sequencing
