# Supplementary material for: Pneumococcal carriage among children under five in Accra, Ghana, five years after the introduction of pneumococcal conjugate vaccine
Source: BMC Pediatr. 2019 Sep 5;19:316. doi: 10.1186/s12887-019-1690-5 (PMC6727402; doi:10.1186/s12887-019-1690-5)
Supplement: Supplementary file 1 — Table S1. The table presents the serotype distribution data from Dayie et al. [13] and the data from the present study, thereby making it possible to compare the data from the two carriage studies. (DOC 118 kb) [file 12887_2019_1690_MOESM1_ESM.doc]

Pneumococcal carriage among children under five in Accra, Ghana, five years after the introduction of pneumococcal conjugate vaccine

Running title: Serotype distribution of *S. pneumoniae* in Ghana

Nicholas T.K.D. Dayie, Dept. of Medical Microbiology, School of Biomedical and Allied Health Sciences University of Ghana, Accra, Ghana. E-mail: nicholasdayie@yahoo.com.

Elizabeth Y. Tettey, Dept. of Medical Microbiology, School of Biomedical and Allied Health Sciences University of Ghana, Accra, Ghana. E-mail: lizteykemgh@yahoo.com.

Mercy J. Newman, Dept. of Medical Microbiology, School of Biomedical and Allied Health Sciences University of Ghana, Accra, Ghana. E-mail: newmerci@yahoo.co.uk.

Elizabeth Bannaman, Dept. of Medical Microbiology, School of Biomedical and Allied Health Sciences University of Ghana, Accra, Ghana. E-mail: na@sonbee.net.

Eric S. Donkor, Dept. of Medical Microbiology, School of Biomedical and Allied Health Sciences University of Ghana, Accra, Ghana. E-mail: ericsdon@hotmail.com.

Kwaku Appiah Labi, Dept. of Medical Microbiology, School of Biomedical and Allied Health Sciences University of Ghana, Accra, Ghana. E-mail: guylabi2@gmail.com.

**Corresponding author:** Hans-Christian Slotved, Ph.D., Senior Scientist, Department of Bacteria, Parasites and Fungi, Statens Serum Institut, Artillerivej 5, DK-2300 Copenhagen, Denmark, Tel: +45 3268 8422, E-mail: [hcs@ssi.dk](mailto:hcs@ssi.dk).

|  | Total number of pneumococcal isolates from Accra (2011) | | Total number of pneumococcal isolate from Accra (2016) | | Serotype included in Vaccines |
| --- | --- | --- | --- | --- | --- |
|  | N = 153 | Percentage | N = 234 | Percentage |  |
| Serotype | All age groups | All age groups | All age groups | All age groups |  |
| NT | 35 | 22.88 | 10 | 4.27 | Non-vaccine serotypes |
| 4 | 1 | 0.65 | 0 | 0.00 | PCV-10, PCV-13, PPV-23 |
| 6B | 15 | 9.80 | 7 | 2.99 | PCV-10, PCV-13, PPV-23 |
| 9V | 2 | 1.31 | 0 | 0.00 | PCV-10, PCV-13, PPV-23 |
| 14 | 8 | 5.23 | 9 | 3.85 | PCV-10, PCV-13, PPV-23 |
| 18C | 0 | 0.00 | 3 | 1.28 | PCV-10, PCV-13, PPV-23 |
| 19F | 23 | 15.03 | 15 | 6.41 | PCV-10, PCV-13, PPV-23 |
| 23F | 8 | 5.23 | 18 | 7.69 | PCV-10, PCV-13, PPV-23 |
| 1 | 0 | 0.00 | 1 | 0.43 | PCV-10, PCV-13, PPV-23 |
| 5 | 0 | 0.00 | 0 | 0.00 | PCV-10, PCV-13, PPV-23 |
| 7F | 1 | 0.65 | 0 | 0.00 | PCV-10, PCV-13, PPV-23 |
| 3 | 6 | 3.92 | 7 | 2.99 | PCV-13, PPV-23 |
| 6A | 8 | 5.23 | 8 | 3.42 | PCV-13 |
| 19A | 2 | 1.31 | 13 | 5.56 | PCV-13, PPV-23 |
| 2 | 0 | 0.00 | 0 | 0.00 | PPV-23 |
| 8 | 2 | 1.31 | 2 | 0.85 | PPV-23 |
| 9N | 2 | 1.31 | 0 | 0.00 | PPV-23 |
| 10A | 0 | 0.00 | 4 | 1.71 | PPV-23 |
| 11A | 4 | 2.61 | 17 | 7.26 | PPV-23 |
| 12F | 0 | 0.00 | 3 | 1.28 | PPV-23 |
| 15B | 2 | 1.31 | 7 | 2.99 | PPV-23 |
| 17F | 1 | 0.65 | 2 | 0.85 | PPV-23 |
| 20 | 1 | 0.65 | 3 | 1.28 | PPV-23 |
| 22F | 0 | 0.00 | 0 | 0.00 | PPV-23 |
| 33F | 0 | 0.00 | 0 | 0.00 | PPV-23 |
| 7C | 0 | 0.00 | 2 | 0.85 | Non-vaccine serotypes |
| 16F | 1 | 0.65 | 23 | 9.83 | Non-vaccine serotypes |
| 34 | 0 | 0.00 | 13 | 5.56 | Non-vaccine serotypes |
| 15A | 0 | 0.00 | 6 | 2.56 | Non-vaccine serotypes |
| 10F | 0 | 0.00 | 2 | 0.85 | Non-vaccine serotypes |
| 19B | 1 | 0.65 | 8 | 3.42 | Non-vaccine serotypes |
| 23B | 1 | 0.65 | 26 | 11.11 | Non-vaccine serotypes |
| 35F | 0 | 0.00 | 1 | 0.43 | Non-vaccine serotypes |
| 13 | 1 | 0.65 | 5 | 2.14 | Non-vaccine serotypes |
| G23 | 0 | 0.00 | 1 | 0.43 | Non-vaccine serotypes |
| 31 | 0 | 0.00 | 3 | 1.28 | Non-vaccine serotypes |
| 38 | 2 | 1.31 | 5 | 2.14 | Non-vaccine serotypes |
| 40 | 0 | 0.00 | 2 | 0.85 | Non-vaccine serotypes |
| 15C | 0 | 0.00 | 3 | 1.28 | Non-vaccine serotypes |
| 18B | 0 | 0.00 | 1 | 0.43 | Non-vaccine serotypes |
| 28F | 2 | 1.31 | 3 | 1.28 | Non-vaccine serotypes |
| 46 | 1 | 0.65 | 1 | 0.43 | Non-vaccine serotypes |
| 6C | 5 | 3.27 | 0 | 0.00 |  |
| 15F | 2 | 1.31 | 0 | 0.00 |  |
| 19C | 8 | 5.23 | 0 | 0.00 |  |
| 23A | 3 | 1.96 | 0 | 0.00 |  |
| 24A | 1 | 0.65 | 0 | 0.00 |  |
| 25F | 1 | 0.65 | 0 | 0.00 |  |
| 36 | 1 | 0.65 | 0 | 0.00 |  |
| 39 | 1 | 0.65 | 0 | 0.00 |  |
| 42 | 1 | 0.65 | 0 | 0.00 |  |

Table S1. The table present the serotype distribution data from Dayie et al [13] and the data from the present study, thereby making it possible to compare the data from the two carriage studies.
